# Psychological drivers in doping: The life-cycle model of performance enhancement

**DOI:** 10.1186/1747-597X-3-7

**Published:** 2008-03-10

**Authors:** Andrea Petróczi, Eugene Aidman

**Affiliations:** 1 Kingston University, Faculty of Science, School of Life Sciences, Penrhyn Road, Kingston upon Thames, Surrey, KT1 2EE, UK; 2 University of Adelaide, School of Psychology, North Terrace Campus, SA 5005, Australia

## Abstract

**Background:**

Performance enhancement (PE) is a natural and essential ingredient of competitive sport. Except for nutritional supplement contamination, accidental use of doping is highly unlikely. It requires deliberation, planning and commitment; and is influenced by a host of protective and risk factors.

**Hypothesis:**

In the course of their career, athletes constantly set goals and make choices regarding the way these goals can be achieved. The cycle of *choice – goal commitment – execution – feedback on goal attainment – goal evaluation/adjustment *has numerous exit points, each providing an opportunity for behaviour change, which may or may not be related to the use of prohibited methods. The interplay between facilitating and inhibiting systemic and personality factors, constantly influenced by situational factors could result in an outcome vector of 'doping attitudes', which combines with subjective norms to influence intentions to choose prohibited PE methods. These influences also vary from one stage of athlete development to the next, making some athletes more vulnerable to engaging in doping practices than others, and more vulnerable at certain time periods – and not others.

**Testing the hypothesis:**

Model-testing requires a series of carefully planned and coordinated studies. Correlational studies can establish relationships where the directionality is not-known or not important. Experimental studies with the manipulation of doping expectancies and risk factors can be used to demonstrate causality and evaluate potential intervention strategies. The final model can be tested via a behavioural simulation, with outcomes compared to those expected from literature precedence or used as a simulated computer game for empirical data collection.

**Implications:**

A hypothesized life-cycle model of PE identifies vulnerability factors across the stages of athlete development with the view of informing the design of anti-doping assessment and intervention. The model suggests that, instead of focusing on the actual engagement in prohibited PE practices, deterrence strategies are likely to be more effective if they target the influencing factors at the appropriate stage and identify groups of athletes and their respective career stages, which pose particular risks of engagement in doping practices. This enables a more effective intervention approach by targeting specific risk factors and expectancies.

## Background

The amateur notions of the 'gentleman sport' have been surpassed by the need for effectiveness and efficiency in order to maintain the constant improvement of sports performance. Activities once unacceptable – e.g. being coached or trained [1] – have become norms. Harold Abrahams' gold in the 1924 Olympics 100 metres sprint stood out for the professionalism of his preparation, which included systematic training and hiring a coach [2], it would be unusual not to take this course of action today. The athlete body is now seen as a highly specialised 'tool' that is altered for maximal performance [3]. Scientists constantly seek ways to improve sports performance. In the early years of training, activities were aimed to refine skills and perfect techniques; the paradigm shift from fixed to expendable capacities, hence to performance enhancement, had not occurred until the cold war era [4]. Athletes today are expected and encouraged to seek every possible way to improve their performance, including specialised training, hi-tech design of equipment and apparel, scientific and medical support, including the use of nutritional supplements [5]. Being a high performing athlete is a profession that requires dedication, long-term commitment and sacrifice [6].

Although there are many ways to put strain on health during the athletic career (e.g. excess training, injuries or disordered eating), the greatest concern to sport governing bodies is the chemical alteration of athletic performance [7,8]. The intriguing question here is what compels athletes to risk their health or reputation for outstanding sports performance and what factors make athletes vulnerable to doping and at which point of their careers.

Among personality factors, low self-esteem and high trait anxiety [9] were found to be contributing factors to doping among preadolescents, but a reversed pattern was observed in high school athletes, where substance users appeared to be less anxious and more self-confident than their non-user counterparts [10].

Engagement in risky behaviours outside the sporting context and using nutritional supplements have been found to increase the likelihood of doping [9,11,12] and self-efficacy in risky situations [12] has been linked to prohibited substance use. In terms of predicting behavioural outcome, attitude and beliefs usually correlate significantly with doping behaviour [12,13] and in many cases, behavioural intention was found to be the strongest predictor [9,12,14]. Strelan and Boeckmann's model of hypothetical doping use posits that personal moral beliefs and health concerns act as preventing factors, whereas drug testing and sanctioning have little deterrent effect [15]. Interestingly, in a search for predicting factors of steroid use, no significant difference was found in the characteristics of the steroid users and those who were offered but reportedly declined the drug [16].

Athletes' self-reported reasons for taking performance enhancing substances are mainly related to achieving better performance or inner desire to win [10,17-26], improving appearance [22,25], perceived external pressure [17,23,25], and fear that competitors have a chemically or medically enhanced, unfair advantage [27-29]. Painkilling drugs and other doping agents are also viewed as a necessary means to overcome injuries [17,26,30-32]. Athletes may also use doping as a means to cope with the extraneous physical demands of training and competition [29]. Whilst most athletes would prefer to compete drug-free, those who are involved in high level sport competitions tend to agree that doping is a necessary add-on to competitive sport [22]. Many athletes are inclined to use doping provided that the drug is undetectable [31,33], while others do not see doping as a 'problem', to them it is a part of their normal training regime [34,35]. Availability or access to performance enhancing drugs is perceived by athletes as a barrier they must overcome if they are determined to use such means [36]. A recent study among adolescent athletes from all levels shows that almost half of the athletes had paid for the drug whereas for approximately 10–14%, the drug was offered by a friend, parent or family doctor [10].

The degree of rationality in doping decision making is highly debated [37]. Economic models of doping mainly assume that athletes act according to economic rationality. The literature in this area [38-42] considers doping as a special case of a *prisoners' dilemma *[43], where one actor's action has consequence for both actors and the best collective strategy is difficult to reach due to lack of information on, and trust for, the other actor's decision. To translate the dilemma into sports, athletes' best case would be to compete at doping-free events. However, the widespread suspicions and speculations about other athletes' possible actions, coupled with the lack of information about the others' doping behaviours have the potential to bias most athletes in favour of doping: game-theoretic modelling suggests that the majority of competitors are likely to see doping as their best option and, under certain circumstances, the only feasible strategy to ensure winning [41].

Whilst economic models of doping have ignored individual dispositions toward doping when it comes to decision making, they emphasise the importance of a broader situational context, within which decisions are not only made on individual preferences but in consideration of others' actions. Existing behavioural doping models have made attempts to incorporate personality, decision making rationality and situational context, including peer group and subculture influences [44-50]. Many of them have touched upon attitudes and other important factors contributing to doping but with a few exceptions [13,18], there has been little attempt at empirical model building or testing.

## The hypothetical model

The model we propose here can be characterised by i) the combination of trait, systemic and situational factors, ii) its developmental approach and iii) the assumed outcome expectancy that led to functional use of PE substances by athletes. A similar approach has been proposed in dealing with substance abuse [51]. The central premise of the model is the dynamics of the PE expectancies. The expectancy theory posits that a given behaviour is motivated by the desire to attain an expected positive outcome and at the same time, controlled by the expected undesirable outcomes from that behaviour [52]. Athletes' motivation to engage in, or refrain from prohibited PE practices is assumed to be influenced by the magnitude and dynamics of positive and negative expectancies and their developmental pathways. Individual differences between athletes and intrapersonal fluctuations across developmental stages are explained by the changes in the PE expectancy construct, which may serve as an effective starting point for anti-doping interventions.

Our model is based on the notion that doping practices grow out of habitual engagement in a range of acceptable performance enhancement (PE) practices, such as physiotherapy, advanced nutrition, training techniques, specialised equipment and apparel [53]. This growth is influenced by two distinct classes of vulnerability factors, controlled by internal and external inhibiting factors and constantly moderated by the situational factors. The model also recognises that drug taking behaviour does not happen in a vacuum. Social, economic, political and cultural environmental constituencies influence people's choices and decisions. Such environmental factors may include i) the legal status and easy access to performance enhancing drugs, ii) medical and pharmacological advancements, iii) political and economic climate, and iv) the general attitude toward using drugs to assist with aspects of life not necessarily require medical treatment (a phenomenon known as medicalisation [54-56]). The increasing medical intervention has been justified on the grounds of the unique needs of a high performing athlete. Athletes rely upon medical help to reach their maximum potential, to prevent injury or shorten recovery time if injury happened [5,57] and such intervention is not only widely accepted but expected by all stakeholders.

The life-cycle model of doping assumes a strategic (or functional) use [58] of performance enhancements. As such, it has been developed for athletes who regularly participate in organized sports competition, where the aim is winning, being the best or setting/breaking a record. However, the life-cycle model of PE and the vulnerability factors are applicable to all sporting and non-sporting situations where a perceived 'pill taking' shortcut or 'quick fix' is available. For example, steroids, growth hormones, stimulants and even diuretics have shown to be used among health club users to improve appearance, rather than athletic performance [59,60]. It has been documented that high school students routinely use supplements and performance enhancing substances for a variety of sport and non-sport related reasons [61,62]. Therefore, the model can be applied to all situations where the end goal (sport performance enhancement) can be substituted with alternative goals (enhancement of appearance, body image, weight loss, etc.). With the exception of diuretics and laxatives that are routinely used as a 'quick fix' to weight problems and often in association with eating disorders [63-67], none of these goals are realistically achievable without work, determination and goal commitment. Despite the marketing claims, chemical assistance alone does not yield sustainable results.

### Influencing factors

**Figure 1:**
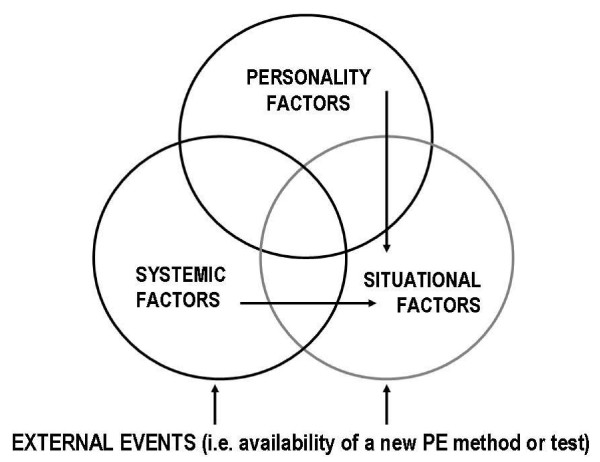
The major groups of vulnerability factors in doping.

Stages of the life cycle model of PE are affected by a host of vulnerability and inhibiting factors, which are categorised as: i) individual differences and ii) systemic factors (Fig 1). The interplay between the relatively stable personality and the arbitrary (hence temporarily stable) systemic factors is constantly influenced by the fluid situational and environmental factors that relate to the ever-increasing pressure to win, perceived behavioural control, availability of doping and alternative PE methods, access to PE drugs, current use of nutritional supplements and prior experience with prohibited PE methods. Shared norms in individuals' social group and their social capital are also thought to play an influencing role in choices regarding PE practices [35]. These contextual contingencies are assumed to moderate the synergy between personality and systemic factors and have effects on the choices athletes made in their PE life-cycle.

#### Vulnerability factors

Investigating predicting factors, a range of psychological variables have been linked to doping behaviours [37]. These individual difference factors include dispositional risk taking and sensation seeking, attitude toward peers, authority and 'fair play', self-esteem, confidence and integrity, cognitive ability, beliefs about doping efficacy, independence and vulnerability to peer pressure.

From athletes' perspective, doping-related risks stem from two distinct sources. First, the current test-based deterrence system poses a risk of failing the doping test, which may be further exaggerated by the governing bodies responsible for anti-doping intervention as a control measure. Second, some PE methods pose considerable health risks to athletes. Unfortunately, under the current anti-doping regulation, it is likely that athletes (and other stakeholders) involved in prohibited performance enhancement practices will place a greater emphasis on avoiding a positive test than on avoiding health risks. In the present punitive system, testing may be perceived by all stakeholders as a barrier to overcome (e.g. to avoid a positive test) and health concerns come only second to using effective but undetectable or not-yet-prohibited (i.e. new or experimental) methods. Anti-doping intervention strategies are likely to be more effective if they incorporate changes in the regulatory system that addresses both health protection and preservation of the 'drug-free' sport principles.

The hypothesised systemic factors include motivational climate, authority structure and PE culture in athletic teams and wider athletic community, as well as perceived fairness and other attributes of the testing procedures and enforcement sanctions. These systemic factors impact on the individual athletes' career progression and opportunities. Competitive athletes naturally strive for achievement, but the ways in which achievement can be accomplished may differ considerably from one individual to another; or from one context to another. It is safe to assume a consistent level of achievement motivation in the population covered by the model: aspiring athletes are all likely to be high achievers [68]. The nature of athletes' achievement striving may take two distinct forms: ego orientation or task orientation, motivated by internalised behavioural norms and desire for personal improvement or winning and external comparison [69]. These orientations are linked to self-beliefs about their own abilities being fixed or incremental [70]. Motivational climate is critical to forming these self-beliefs and subsequently, the task-vs-ego orientation. Anti-doping interventions that focus on developing mastery climate and foster task orientation in sport-related goals are likely to reduce athletes' inclination to use unacceptable PE methods.

Motivational climate is shaped by external achievement expectations from coaches, parents, peers and fans, as it is perceived by the athlete. Whether the athlete perceives the achievement expectation from his/her environment as a progress and constant improvement (so called 'mastery climate') or as constant competition and desire to win ('performance climate') influences the athlete's subsequent choices and behaviours [71]. An emphasis on the outcome, such as results and winning, is more likely to lead to maladaptive motivational and affective responses such as taking short-cuts, cheating or aggressiveness [72]. Furthermore, the PE subculture of a particular sport may prompt athletes to take drugs in order to show solidarity with peers, or to enhance their (athletic) identity by engaging in doping practices which are seen by the subculture as transitional markers denoting the transition from 'amateur' to being a serious athlete [46]. Altering role expectancies (i.e. what it means to be a high-performing, serious athlete) are likely to be particularly effective in interventions with adolescent athletes in sports with high prevalence of doping.

#### Inhibiting factors

Factors that may avert individuals from using performance enhancing drugs are related to the current punitive-sanctioning anti-doping system, cultural – religious norms, moral values, social pressure from close relatives and friends and health concerns. Some personality traits can act as inhibitors of doping engagement (e.g. positive and stable self-esteem, conscientiousness and low risk-taking propensity). Intervention as a preventive factor has been the most extensively researched aspect. Gender specific, team centred education has been shown to result in self-reported behaviour change in the Adolescents Training and Learning to Avoid Steroids (ATLAS) [73,74] and the Athletes Targeting Healthy Exercise and Nutrition Alternatives (ATHENA) [74,75] but effectiveness of such education programmes should be validated beyond self-reports.

Athletes may also refrain from doping or using other supplements if they perceive the consequences of a positive doping test as unacceptably high. However, while a random drug testing program (SATURN) among high school athletes did not result in significant reduction in prevalence, it has increased some risk factors (reduced belief in own athletic ability and more permissive attitude) for future substance use [76]. Furthermore, drug testing as an inhibitor does not have any relevance to those who use performance enhancing supplements outside sporting arena.

#### Situational factors

**Figure 2:**
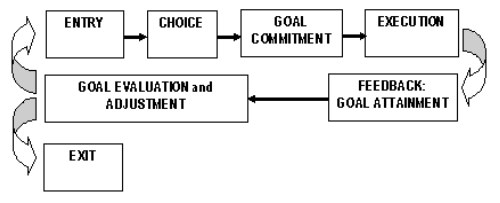
Life cycle model of performance enhancement.

The hypothesised situational factors include the dynamics of peer interactions [44], salience of role models and significant others [45] and, most importantly, the availability of performance enhancement alternatives – both acceptable and illegal [37]. Most of these factors have a strong developmental aspect to them – as attitudes, role models, vulnerability to peer pressure, performance enhancement motivation and the ingredients of moral choice tend to change systematically form one stage of athlete's development to the next. This multitude of influencing factors result in an outcome vector called 'doping attitudes', which, according to the Theory of Reasoned Action [77] and Theory of Planned Behaviour [78], combine with subjective norms to influence intentions to choose doping at one stage of the PE life-cycle. The dynamics of this process is demonstrated in the life-cycle model of PE (Fig 2). Transition points providing opportunities for interaction are therefore to be expected, with some of these factors gaining – and others loosing – their salience and impact. Exploiting the interactions between personality traits, behavioural tendencies and doping expectancies is likely to improve the effectiveness of intervention and deterrence strategies. For instance, research in substance abuse shows that expectancies have a greater influence on impulsive than non-impulsive individuals [51].

#### Environmental factors

Environmental factors include the socio-cultural, political milieu, the legislative system, as well as the availability of drugs, new drug discoveries and permitted alternatives, such as nutritional supplements, minerals, herbs and non-herb non-mineral substances. The importance placed on sporting success in a society and the direct and indirect reward for such success influence the motivational climate in which athletes prepare and compete. Criminalisation or decriminalisation of the PE drugs may only have an effect on the level of use via the distribution mechanism. Studies show that decriminalisation of social drugs do not have the effect on the prevalence rate [79,80]. The situation with PE drugs is more complicated as the array of PE practices includes drugs and methods that require medical assistance and freely available supplements that may or may not be on the Prohibited List but have similar ergogenic properties to the prohibited drugs [81,82]. The widespread medicalisation of life creates an environment in which using medical or chemical assistance to life is widely accepted and normal. Many athletes believe that they need assistance in order to cope with the physical demand of training, workload, injury and recovery. Under the current anti-doping regulation, athletes may turn to PE methods and substances that are not included in the Prohibited List in order to: i) eliminate the risk of being caught, ii) play by the rules or iii) conform to the ethical or moral principles imposed on them by their social environment. However, the potentially negative impact on health [83,84] should be of particular interest as the health risks associated with nutritional supplements are often overlooked or minimised. The proposed alternative testing methods (i.e. biological passport or health passport) where the ergogenic effects or unexplained changes in biological markers are detected instead of the presence of certain drugs in the body may address this gap, hence the effect of these proposed regulatory changes should also be considered among the environmental factors.

### The life-cycle model of performance enhancement

The use of performance enhancing methods is unlikely to be an accident. It requires sustained, self-regulated, goal-directed effort [85,86]. Doping use is assumed to exhibit similar characteristics to 'functional drug use' which has been recognised as a specific from of drug use [87,88]. Functional drug use is distinct from experimental, recreational or dependent use (abuse/addiction) and it refers to a strategic use of substance to achieve a set goal (i.e. improve a function or skill). Examples for functional use include but are not limited to using stimulants to increase alertness or balance long working hours; or taking hypnosedatives to help coping with anxiety, stress or depression [58]. Functional drug use is not necessarily problematic in the sense of addiction although physical and psychological dependence may develop from functional use. Athletes' reasons for taking performance enhancing substances (as discussed earlier) are in keeping with the definition of functional drug use. One of the main advantages of the life-cycle model over the traditionally used trait or situational models is that the developmental nature of the proposed model offers various intervention points and suggests a varying set of methods to match those points. These intervention strategies are discussed in detail along with the description of the life-cycle model of performance enhancement and can be summarised as follows: Chronologically, the first intervention points focus on the risk factors by preventing the onset of risks or transforming risks that are already present. Whilst these options are considered less effective in substance abuse [51], the unique nature of performance enhancing substance use offers scope for intervention at this stage by changing not the athlete but the environment that influences the onset of doping use. With that in view, the responsibility for deterrence is broadened from individual athletes to the inter-related system of rules, regulations, expectations by coaches, support personnel and policy makers. An intervention approach that aims to alter expectancy trajectories or modify current maladaptive expectancies (e.g. in relation to steroid use which is prone to developing physiological and psychological dependence) is likely to produce more lasting effects. Based on research on substance abuse [51], it is envisaged that preventing the development of positive doping expectancies before the onset of a doping-related event may work well with pre-adolescents, whereas athletes with doping experience or in positions to seriously contemplate the use of prohibited PE methods may respond better to modifications of expectancy pathways, especially if comparable and acceptable alternatives are offered.

Our model is based on the existing models of self-regulation containing a number of stages by which athletes engage in performance enhancing practices. These include the phases of: i) choice, ii) goal commitment, iii) execution, iv) goal attainment feedback, v) goal evaluation and adjustment and vi) decision to continue (start the next cycle) or exit. Each phase of the cycle has its risks and vulnerabilities. They contain breaking points that can be exploited by a carefully designed anti-doping intervention (Table 1).

**Table 1:** Vulnerability factors and potential individual and systemic remedies

	**Potential remedies**	
**Vulnerability**	**Individual (therapy style, one on one intervention)**	**Systemic (system level adjustment)**

Compulsiveness	Perspective taking, balanced life goals	Athletes as a person, not result-generating device
Single mindedness	Self-esteem work: winner vs. worthwhile, self-respecting person	Career transition (i.e. life after sport)
Risk aversion	Communicate the risk: health, legal, financial, social, psychological	Support independent research re toxicity of PE methods to established standard
Expediency	Value system, collaborative work	Replace prohibition with mandatory informed consent and supervision Incentives for fair play

#### Choice

The cycle starts with an entry point during the athlete's career when specific (typically short to mid term) performance target is set, either by the athlete or their coach, and a means of performance enhancement is chosen to service that target. The key question of this phase is: *'What is the end you want to achieve?' *The range of motives for engaging in coordinated performance enhancing practices can be classified into one of the three main types. First, there is the achievement motivation, the drive to get ahead of others in the sense of winning and record breaking aspirations. Second, there are affiliation motives that are based on the need to be liked and lead to seeking fame and popularity. Finally, intrinsic motivation leads are present to seeking satisfaction from the chosen activity itself, i.e. from high intensity training. The choice outcomes of this phase are likely to be influenced by the combination of i) costs (including time and effort) and availability of the method(s), and ii) personality factors, such as compulsiveness or single mindedness [44]. The potential intervention at the individual level might involve teaching perspective taking, multiple goal setting and developing a broader perspective on life beyond sport. These intervention strategies can (and should) be complemented by compelling other stakeholders, such as parents, coaches, sport organisations, society and media to support and encourage the athletes' broader goals. Doping expectancy alterations can be achieved by modifying associations with positive outcomes, e.g., by providing healthier and more acceptable alternatives while reinforcing the negative associations of doping with health consequences.

#### Goal commitment

Achieving the gains of performance enhancement requires considerable commitment and hard work from the athlete. PE objectives cannot be achieved on a whim. They require sustained effort and self-discipline over a considerable period of time. The key question of this stage is: *'How badly do you want it?' *A strong goal commitment becomes a necessary entry requirement at this stage of the life-cycle model of PE. It may flow naturally and implicitly from the athlete's passion for their sport based on any combination of achievement, affiliation or intrinsic motives described above. It may also take the form of 'conscious assembly of reasons' when athletes deliberately search for additional sources of motivation [89]. Interventions may emphasize the scope and complexity of the undertaking, as well as prompting broader motives for consideration at the 'motivational build-up' stage.

#### Execution

Performance enhancement only provides a time-shortcut. The gains from investment in PE methods depend on the increased density and intensity of training regime. This may also require adherence to new, more complex training regimes to be effective. The key intervention question of this phase is:*'Do you know what is involved and are you prepared for the hard work?' *Following through and executing the intention becomes a distinct challenge. In addition to strong commitment, knowledge and discipline are required to meet this challenge. Support from training and medical personnel and family is likely to play an important role in execution, therefore anti-doping deterrence should incorporate these supporting roles as well. Educating and changing expectancies of family and support personnel has the potential to alter the likelihood of the successful execution of the PE cycle.

#### Goal attainment feedback

During the feedback stage, achieved results are compared to the set achievement goals in order to assess whether the chosen PE method delivered its promises. At this stage, doping expectancies are either confirmed or refuted. Discrepancies between goals and achievements are scrutinised to identify inhibiting factors (i.e. flaws in implementation or falling for false promises). Costs and alternative methods are also considered. The key question at this stage is: *'Have you got what you had hoped for?'*

#### Goal evaluation and adjustment

Sustained self-regulation assumes continuous re-evaluation of goals. Any incongruence between goals and achieved results is examined at with the intention of identifying hindering or facilitating factors and contingencies. The key question at this stage is: *'Has your PE plan worked?' *Conclusions from this stage determine the decision regarding the next stage of the PE life-cycle. This stage offers a good opportunity for anti-doping intervention targeting doping expectancies. Athletes at this stage may have already experienced side effects. It is also plausible that the PE regime worked in terms of improved sports performance but failed to yield the ultimate prize of winning or gaining the expected rewards. Intervention strategies at this stage should provide acceptable and comparable alternatives to prohibited PE methods. Athletes who are not entirely satisfied with their chosen PE regime are likely to be open to modification of doping expectancies.

#### Entry vs. exit of the cycle

The key question of this stage is: '*What is next*?' The outcomes of the goal attainment and the goal evaluation stages lead to one of the three possible outcomes: i) repeating the cycle without changes, ii) repeating the cycle with modification, and iii) abandon the effort of performance enhancement.

#### Summary of the life-cycle model

There is no reason to expect fundamental differences in the cognitive or motivational process involved in the athlete's decision cycle whether it deals with acceptable PE or doping methods [90]. Hence, the model of PE presented above does not make a distinction between accepted and prohibited means of PE. Both need a sustained, motivated, goal-directed action. The difference is brought about by the current convention of the sport, in particular whether or not the method is deemed to be acceptable or unacceptable in it.

## Testing the hypothesis

Considering the complexity and reiterative nature of the model, empirical testing of the model as a whole is not feasible. Hence it is proposed that the robustness of the model should be tested via a series of simulated cases where the behavioural outcomes are compared to those expected from literature precedence or prior empirical studies. Decisions made in hypothetical situations have been shown to be predictive of relevant behaviour [15].

### Proposed Approach

In order to set the parameters of the model, knowledge can be accumulated via a thorough literature search where scientific evidence is available for i) drug efficacy, detectability and toxicity, ii) comorbidity of drug and doping [91-94]; nutritional supplements and anabolic steroids or other doping [11,90,95], as well as iii) relevant personality factors and their influence on behavioural outcomes [12,14,15,49,50].

A series of independent studies should aim at gaining empirical evidence regarding the influence of each identified factor on the respective elements of the model. Correlational studies can establish relationships where the directionality is not known or of no importance. Experimental studies with the manipulation of doping expectancies and risk factors can be used to examine causality and evaluate potential intervention strategies. Specifically, empirical research contributing to the model parameters could be conducted in one or a combination of the following areas:

i) Experimenting with and developing reliable methods for collecting information on or estimate the prevalence of doping behaviour (i.e. implicit assessment or estimation);

ii) Identifying internal risk and protective factors;

a. personality factors that are linked to doping behaviour (e.g. achievement orientation, self-belief, self-esteem, self-efficacy, trait anxiety, risk taking/aversion, etc.);

b. relationship between explicit and implicit attitude and investigating their effect on behaviour/decision making;

c. moral value and belief system, knowledge and health beliefs;

iii) Identifying external risk and protective factors;

a. environment effect (e.g. socioeconomic variables, type of sport, motivational climate, etc.)

b. influence of close others (e.g. family, coach, peers, doctors, etc.);

c. effect of the current and alternative anti-doping system (e.g. punitive-sanctioning vs. permissive-controlling) on decisions regarding doping use;

iv) Establishing the likelihood of/odds ratio for using doping from various situational factors (e.g. nutritional supplement use, type of sport, vested interest, opponents' known or perceived actions, etc.)

Results from the independent studies can be combined into the life-cycle model of performance enhancement, which can be tested via researcher-led simulations by running series of hypothetical scenarios with different combinations of personality, systemic, situational and environmental factors. The model can also be used for empirical testing, with being presented as a computerised strategy game to athlete participants. The ultimate goal of the game would be to maximise achievement within a given timeframe.

One of its key advantages of measuring complex cognitive functioning through synthetic environments and simulations is that it is indirect, unobtrusive and entertaining. The potential of computer game-embedded measurement methodology in the assessment of personality and social behaviour is in its capacity to minimise self-presentation effects in the assessment of individual choice in contexts of potential inter- and intra-personal conflict [96,97]. The value of such an approach is re-affirmed by the prospects of psychological testing in virtual reality where interpersonal interaction is cited among the most immediate and attractive assessment targets [98]. Systematic observation of player response to simulated environments in synthetic but realistic performance enhancement scenarios has the capacity to take the measurement of intended doping-related behaviour beyond the inevitable distortions of self-report methodology – whether these distortions are intentional or not. Such observations alleviate the limitations of the experimental models, while retaining the advantage of being safe and controlled.

The impact of systemic factors on intention formation can be examined both directly and in interaction with relevant personality factors (i.e. risk aversion and expediency) within experimental research frameworks. Athletes can be presented with an option to take a new drug in a hypothetical situation and asked to rate their intention to use. Risk aversion would be expected to interact with toxicity and expedience with detectability in predicting the strength of implementation intention. Similar predictions can be made for other phases of the model. Drug detectability, availability, efficacy and toxicity can be manipulated in a between-subject design, where respondents belong to one of the known 'personality' groups (i.e. goal/task oriented, risk aversive/risk taker). Hypothetical scenarios may also include new approaches to anti-doping prevention such as the 'white list' (legalised performance enhancing substances), biological or health passports to investigate the likelihood of their inhibiting effect. Whether different moral and ethical values are applied to different situation on and off the sporting field may also merit future investigation in order to establish the presence or absence of a 'carry over effect' between sport (bounded by rules and aimed at scarce resources) and other spheres of athletes' lives. This 'carry over effect' may also be an important aspect of performance enhancing drug use by a non-athlete population.

A relationship between social drug use and doping has been shown in most relevant studies [10,99-101] suggesting that the use of social drugs increases the chance for doping and vice versa. However as most of these studies used self-reported information, such claim should be interpreted with caution and the phenomenon should be investigated further by using alternative methods. For example, self-report data on taking performance enhancements using Random Response Technique (RRT) yield a considerably higher percentage of athletes admitting doping than direct anonymous self-reports did [102,103]. Underscoring the fundamental shift from individual characteristics of a performance enhancing drug user to peer influences, the social network approach has been successfully applied in research regarding substance use and designing, implementing and evaluating effective prevention programmes [35,104]. Individual drug use (or non-use) is strongly associated with the same behaviour in the immediate social circle. Therefore, network level characteristics such as density or centrality might be used as a predictive or inhibiting factor in substance use [104].

## Implications

The current anti-doping policy has received much criticism for its elite focus, sanction-based approach and associated costs [53,105-108]. However, the growing number of educational programmes are designed and implemented by the sport governing bodies to target varying groups from top performing athletes to young talents focus on the principles of performance enhancement and fair play.

In contrast, the model presented here focuses on vulnerability factors across the stages of athlete development enables us to i) develop empirically testable developmental hypotheses and ii) examine the moderating impact of subjective norms and motivational climate on athletes' intentions to engage in doping. The simultaneous consideration of risk factors (facilitators) and protective factors (inhibitors) is a generally accepted approach in dealing with delinquent behaviour, such as drug use, drinking, violence or crime [109]. Establishing a sport-specific set of facilitators and inhibitors (and their interaction) is pivotal to anti-doping prevention. Whether the elimination of performance enhancing drugs or harm-reduction is the intended outcome, the conundrum of performance enhancement must be overcome before meaningful prevention strategies can be designed and implemented. The proposed model offers a framework for a promising deconvolution approach to doping as well as for performance enhancing drug use prevention in the non-athlete population.

The model may be used as a framework for the design of anti-doping assessment and intervention methods. Instead of focusing on the doping attitude (outcome of the combination of the vulnerability factors), the model encourages an alternative of proactive consideration. In preference to the costly and challenging detection-based approach [108,110], which sanctions and penalises individual athletes if the presence of the drug in their system (regardless of the effect) is indicated or with evidence of involvement in doping practice; we suggest targeting the influencing factors that lead to doping. A holistic approach with the emphasis on choices, health issues and broader life goals to the individual and the systemic factors is needed in order for athletes to make informed decisions about their performance enhancement, which may lead – at the population level – to a sustainable change in doping behaviour. Overall, anti-doping interventions are likely to benefit from a dual focus on the risk factors and doping expectancies, as well as from targeting the athlete population from preadolescents to adults at all stages of PE life-cycle.

## Competing interests

The author(s) declare that they have no competing interests.

## Authors' contributions

AP reviewed the literature, investigated the situational and systemic factors and drafted the manuscript. EA developed the vulnerability cycle model, added considerations of psychological factors and contributed to drafting the manuscript. All authors have read and approved the final version of the manuscript.
